# Perspective: ethical considerations of animal-sourced foods

**DOI:** 10.1093/af/vfae036

**Published:** 2025-04-05

**Authors:** Paul B Thompson

**Affiliations:** Departments of Philosophy, of Community Sustainability and Agricultural, Food and Resource Economics, Michigan State University, East Lansing, MI 48824, USA

**Keywords:** agricultural ethics, agrarian philosophy, sustainable intensification, technological ethics

**Implications**

- The institutional capacity for ethical research in agriculture is underdeveloped.
- Mainstream technological ethics emphasizes innovations that promote improvements in health, wealth, and well-being, but constrains technology that does this at the expense of individual rights and vulnerable populations.
- Other philosophical traditions prioritize the ethical importance of producing food, as well as investing in food producers themselves with unique cultural and ethical values. They introduce important elements into agricultural ethics.

## Introduction

Technological ethics is an organized multidisciplinary research activity incorporating theory and methods from philosophical ethics, the law, and the social sciences along with technical expertise from respective STEM fields. Ethical analysis of STEM research encompasses the critical examination of the rationales for prioritizing research streams, as well as anticipating and evaluating the health, safety, and environmental impacts of innovations. Technological ethics often involves an exchange of views on cultural and socioeconomic values that both support and militate against pursuing any given course of change in technical practice. Such methods have been institutionalized under headings such as Ethical, Legal, and Social Issues (ELSI) at agencies including the U.S. National Institutes of Health, and as Responsible Innovation in many engineering fields and throughout Europe. Agricultural institutions have been slow to develop a capacity for ethical analyses to accompany a technical research process. While U.S. medical schools have entire departments dedicated to bioethics, few agricultural universities, and research institutes include staff with the philosophical, legal, and humanistic training that has become central to the bioethics practiced in a medical research context (Zimdahl 2022; Kendig *et al.*, 2024).

With agriculture’s weak capacity for ethical analysis in mind, this perspective piece introduces four overarching frameworks, or archetypes that articulate distinct philosophical approaches to the ethics of agricultural innovations. Each draws upon implicit understandings of what agriculture—including animal agriculture—is and its role in providing ethically important goods. The *technological modernization* ethic has its origins in the industrial revolution and stresses the role of technical change in humanity’s enormous gains in health and material well-being over the last 250 years. At the same time, the uneven distribution of these benefits is viewed as ethically problematic. It summarizes the approach most widely taken in nonagricultural STEM fields. At the same time, agriculture differs from other fields both in the moral imperatives that derive from its focus on a basic human need, as well as from philosophical traditions that extend into ancient times. Three versions of specialized agricultural ethics thus compete with technological modernization: *sustainable intensification; urban agriculture* and *rural resilience.*

## Technological Modernization and the Food System

In general, industrial methods reduced the cost of manufactured goods, making them available to a much larger swath of the total population. Utilitarian ethical philosophy emerged along with industrialization, supporting social changes of all kinds when they improve the quality of life for a larger share of the population. On the one hand, production efficiencies that reduced the cost of household goods (including food) were especially compelling because they tended to produce proportionately larger benefits for those on limited incomes. This view is broadly utilitarian: technological change is ethically justified because it tends to produce “the greatest good for the greatest number,” On the other hand, appeals to human rights and social justice were intended to insure that the costs of technological progress are borne fairly. Principles of fairness may challenge utilitarianism’s assumption that benefits are always able to offset harm to third parties. They stress equality and power sharing, or hold that one person should not be allowed to impose costs on others without their consent (Vallor, Raicu, and Green 2022).

There is a fundamental tension between these two norms, exemplified in several mid-20th century instances of biomedical research. Most notoriously, researchers conducting a longitudinal study of syphilis in Tuskegee, AL declined to offer treatments to research subjects even after penicillin was recognized as an effective cure. Their reasoning concluded that the social benefit of a more detailed understanding of the etiology of the disease outweighed the harm to a few individuals enrolled in their study. Although racial bias also played a role, the key point for present purposes is the tension between a rule or norm that rationalizes research in terms of its risks and benefits and one where individuals retain the right to choose whether they will participate. The Tuskegee study was a precipitating event in the U.S. National Institutes of Health’s creation of consent-based standards for research involving human subjects.

The tension between a utilitarian ethic of optimizing costs and benefits and a rights orientation can be seen in recent debates over food labels. A utilitarian view recognizes the cost of product labeling and demands these costs be repaid in terms of social benefit. Health and ingredient labeling can meet this test, but process labels with no discernable differences in the end product do not. Clearly, others have seen it differently, insisting on a consumer “right to know”. Failing to secure this right deprives consumers of the ability to apply personal values in food choice; it frustrates their ability to withhold consent.

I have oversimplified these examples in order to make two points. First, technological changes that produce more benefit than cost can be justified by a moral logic that tells us to achieve the greatest good for the greatest number, but this norm will be challenged when benefits and risks are unfairly distributed or coercively imposed upon parties who have no power to resist them. Second, nothing about this reasoning is peculiar to agriculture and food. Similar themes occur in medical technology, in information technology, and in energy technology. Agriculture is viewed as a sector in an industrial economy, and innovations in any sector will encounter the tension between an outcome-oriented approach to ethics and norms that stress rights, the ability to give or withhold consent, and the imposition of power.

## Ethics in Agriculture

In contrast, ethical traditions for valuing elements and aspects of farming and pastoralism extend deeply into the past. They provide approaches sensitive to ways in which agriculture differs from innovation in other STEM fields. At least three lines of thought can be identified. First, there is the “feed the world” ethic of sustainable intensification. Confidence in this ethic leads innovators to presume that insuring the global capacity for food production for future generations supersedes other moral considerations. Others similarly focused on the overriding importance of food take a less global approach I will characterize as *urban agriculture.* Here the focus is on deficiencies in present-day agriculture. Finally, there is an even older agrarian tradition focused on the moral significance of the farmers themselves. I will discuss this under the heading of *rural resilience.*

Sustainable intensification prioritizes a responsibility to future generations. It is consistent with the 1987 statement of the World Commission on Environment and Development, which states that the needs of the present must not compromise future generations’ ability to meet their own needs. This is arguably the view that prevails among the agricultural science community, who note that both population growth and climate change are predicted to create a global shortfall in the food needs of the global population by the end of the 21st century. Urban agriculture differs from sustainable intensification in two important respects. First, moral considerations are oriented locally, rather than globally. Second, obligations to present-day people are prioritized over future generations. The central ethical commitments of this perspective derive from the judgment that our current food system is failing its moral responsibilities. Those who take this perspective split between a pro-technology group favoring innovations to make cities less dependent on rurally based food production, including synthetic meats and vertical farming, and those who emphasize social tools for strengthening networks among vulnerable populations.

The final category presumes that farmers themselves are the source of agriculture’s special moral significance. Primary agricultural producers are said to deserve special recognition and support. Farmer-oriented ethics were commonplace in the past, but the rationales for them have become obscure and in some cases, obsolete. However, certain dishes or foodstuffs are thought crucial to maintaining a social identity in some urban outlooks. In some instances, the farm household (or a certain configuration of the farm household) serves a symbolic role in reproducing ethnic, regional, and national identities. For example, the veneration of farmers unites otherwise diverse Asian philosophical traditions. Humanity is seen as occupying a middle position between the material world and an ethereal or spiritual plane of ideas and ideal forms. Farming, including animal husbandry, locates humanity’s proper positon in this cosmology (Xu 2018). Philosophies that idealize the farm household may have even greater resonance in less industrialized areas.

## Conclusion

Sustainable intensification prioritizes innovations in light of a moral obligation to insure that future generations can meet their food needs. Urban agriculture evaluates the food system in terms of present-day needs and is motivated strongly by evident failures in the current food system. The rural resilience archetype highlights the needs of the farming population, yet many people who have no direct connection to agriculture are attracted to the rural resilience ethic. Each of these three archetypes attributes special moral significance to agriculture and food systems. They influence food purchases and motivate political activity.

Importantly, people deeply committed to one of these archetypes may have little tolerance for others, especially when they believe their values are being ignored. For example, in 1985, economist Robert D., Kalter predicted that recombinant bovine somatotropin would cause widespread bankruptcy and transformation in the dairy industry. Assumptions in Kalter’s model were disputed by other economists, but the initial debate grew increasingly convoluted and acrimonious, with the U.S. Congress stepping in to enact a one-year moratorium on rBST even after approval by the Food and Drug Administration (FDA). It is at least arguable that the rBST debate set the stage for public skepticism over genetically engineered crops and livestock, and it is doubtful that the debate would have captured the public’s attention if not for a vocal constituency opposing the drug because of its alleged impact on dairy farmers (Thompson 2017).

Ethical analysis of innovations in animal agriculture must begin with the tension created by these three archetypes, but it must also take cognizance of the fact that many do not see agriculture as involving any ethically unique features, in the first place. Technological modernization views animal agriculture as one sub-sector in an industrial economy consisting of firms that compete on many levels. Here, the animal production platform will be allocated according to its most socially efficacious use, subject only to the criterion that injury or harm to third parties (including workers and animals) are internalized. I believe technological modernization has become the dominant way that many people look at agriculture. They are no longer persuaded that agriculture has duties or purposes that justify exceptions to society’s moral expectations for any other sector in a complex industrial economy. This creates a unique challenge for ethical inquiry in agriculture, presenting those who see agriculture as having unique moral objectives with an even greater reason to articulate their value commitments clearly.

## About the Author

Paul B. Thompson is Professor Emeritus at Michigan State University, where he was the inaugural occupant of the W.K. Kellogg Chair in Agricultural, Food and Community Ethics between 2003 and 2022. Thompson received his PhD in philosophy from Stony Brook University. Prior to joining the faculty at Michigan State, he held joint positions between philosophy and the college of agriculture at Texas A&M University and Purdue University. In addition to edited and single author books, Thompson has published more than 250 articles in refereed journals and edited books. His book *From Field to Fork: Food Ethics for Everyone* was reviewed in *Science* and received the North American Society for Social Philosophy’s prestigious “Book of the Year” award for 2015. *From Silo to Spoon: Local and Global Food Ethics* was published by Oxford University Press in 2024. **Corresponding author**: thomp649@msu.edu
